# Gene drive inhibition by the anti-CRISPR proteins AcrIIA2 and AcrIIA4 in *Saccharomyces cerevisiae*

**DOI:** 10.1099/mic.0.000635

**Published:** 2018-02-28

**Authors:** Erianna M. Basgall, Samantha C. Goetting, Megan E. Goeckel, Rachael M. Giersch, Emily Roggenkamp, Madison N. Schrock, Megan Halloran, Gregory C. Finnigan

**Affiliations:** ^1^​ Department of Biochemistry and Molecular Biophysics, 141 Chalmers Hall, Kansas State University, Manhattan, KS 66506, USA; ^2^​ Department of Biology, 116 Ackert Hall, Kansas State University, Manhattan, KS 66506, USA

**Keywords:** CRISPR, Cas9, gene drive, sgRNA, regulating gene drives, biotechnology, anti-CRISPR

## Abstract

Given the widespread use and application of the clustered regularly interspaced short palindromic repeats (CRISPR)/Cas gene editing system across many fields, a major focus has been the development, engineering and discovery of molecular means to precisely control and regulate the enzymatic function of the Cas9 nuclease. To date, a variety of Cas9 variants and fusion assemblies have been proposed to provide temporally inducible and spatially controlled editing functions. The discovery of a new class of ‘anti-CRISPR’ proteins, evolved from bacteriophage in response to the prokaryotic nuclease-based immune system, provides a new platform for control over genomic editing. One Cas9-based application of interest to the field of population control is that of the ‘gene drive’. Here, we demonstrate use of the AcrIIA2 and AcrIIA4 proteins to inhibit active gene drive systems in budding yeast. Furthermore, an unbiased mutational scan reveals that titration of Cas9 inhibition may be possible by modification of the anti-CRISPR primary sequence.

## Introduction

The recent discovery of the clustered regularly interspaced short palindromic repeats (CRISPR) system in prokaryotes [1] has led to a major shift in many fields of molecular biology and biotechnology [2–4]. The ability to introduce a precise genomic alteration (usually, but not always, in the form of a targeted double-stranded break), coupled with evolved DNA repair systems, has allowed for insertion, deletion and modification of genomic sequence in many living cell types and organisms [5–7]. Current applications include combating human genetic disease, pathogens, bioenergy, agriculture, basic biological research and other biomedical uses [5, 8–10].

One particular arrangement of the Cas9 nuclease editing system has been recognized for its potential for pest management – namely, the ‘gene drive’. This genetic system has demonstrated the potential to combat insect-borne diseases such as malaria for its ability to rapidly impose population control through a *super-Mendelian* mechanism [11–15]. Briefly, the Cas9 gene and its corresponding single guide (sgRNA) expression cassette are integrated at or in place of an endogenous locus; the nuclease is targeted to the WT copy of the deleted (or modified) native gene on the homologous chromosome within a diploid cell. Homology directed repair occurs to fix the break and copy the entirety of the gene drive cassette, thus ‘forcing’ a homozygous state and ensuring rapid propagation through a population. There are still challenging technical (and ethical) hurdles facing the development and implementation of gene drive systems including (i) evolved resistance to artificial drive systems in native populations [14–18], (ii) biosecurity [19, 20] and (iii) public approval [21, 22].

A new class of ‘anti-CRISPR’ peptides have been identified – one that evolved within bacteriophages as a direct inhibitor of the Cas9 nuclease [23, 24]. The crystal structure of the AcrIIA4 protein bound to sgRNA-loaded *S. pyogenes* Cas9 illustrates that this class of proteins acts as a DNA mimic to associate with the nuclease where the protospacer adjacent motif (PAM) sequence normally resides [25, 26]. There has been continued interest in the development and application of regulatable Cas9 variants sensitive to small molecules, temperature or external stimuli such as light [27–33]. The anti-CRISPR proteins offer an expanded set of options as a natural ‘off-switch’ for the nuclease for genomic editing [34]. Here, we demonstrate the use of the AcrIIA2 and AcrIIA4 anti-CRISPR proteins as potent inhibitors of a gene drive system in *S. cerevisiae*. Moreover, a comprehensive mutational analysis of both proteins has revealed the potential for a *tunable* level of Cas9 control.

## Methods

### Yeast strains and plasmids

*Saccharomyces cerevisiae* strains can be found in Table S1 (available in the online version of this article). Standard molecular biology techniques were used to generate all constructs [35]. Strains containing Cas9 were constructed by first creating a *CEN-*based plasmid including *HIS3* UTR sequence, artificial [u2] sequences [36], and the Kan^R^ cassette [37] using *in vivo* plasmid assembly [38]. The entire cassette was PCR-amplified with a high-fidelity polymerase (KOD Hot Start, EMD Millipore) and transformed into yeast using a lithium acetate protocol [39, 40]. Diagnostic PCRs followed by Sanger DNA sequencing (Genscript) confirmed successful integration.

DNA plasmids generated in this study are in Table S2. Vectors for the AcrII genes were constructed by *in vivo* assembly under control of pr*CDC11* and *ADH1*(t). For all substitutions, the AcrIIA2 and AcrIIA4 expression cassettes were amplified, cloned into a TOPO II vector (pCR-Blunt II-TOPO, Invitrogen), mutagenized by PCR, and sub-cloned to pRS316 using flanking *NotI/SpeI* restriction sites. Plasmids containing sgRNA cassettes included pr*SNR52* and *SUP4*(t) sequences [41]. All vectors were confirmed via DNA sequencing.

### Culture conditions

Yeast were grown on solid or in liquid medium including YPD (2 % peptone, 1 % yeast extract, 2 % dextrose) or synthetic media (nitrogen base, ammonium sulfate and amino acids). Pre-induction medium included 2 % raffinose and 0.2 % sucrose. Induction (pr*GAL1/10*) media included 2 % galactose. All sugars were filter sterilized.

### CRISPR/Cas9-based editing

Editing of haploid yeast utilized the multiplexing of Cas9 at artificial loci (mCAL) system [36]. Multiplexing of Cas9 was accomplished by programming two DNA sequences flanking a locus of interest with a maximum mismatch to the genome. These artificial targets [u1]/[u2] also included a PAM. Editing was performed by transforming the pRS316-based plasmid (empty or harbouring the AcrII gene) into yeast followed by a second transformation event to add the sgRNA plasmid. Activation of Cas9 was performed as previously described [36, 40, 42]. Briefly, cultures were grown overnight in pre-induction medium, back-diluted into rich medium containing galactose for 4.5 h, transformed with equimolar amounts of the guide plasmid, recovered overnight in YPGal and plated to SD-URA-LEU medium. Surviving colonies were quantified using a single-blind protocol and sectoring method.

### Gene drives and containment

First, gene drive strains were transformed with the *LEU2-*based sgRNA plasmid and a *URA3*-based plasmid (empty or expressing AcrII gene). Second, haploid yeasts were mated with strains of the opposite mating type for 24 h and transferred to diploid selection plates (SD-URA-LEU-HIS) for three rounds of growth. Third, diploids were cultured in pre-induction media overnight, back-diluted into YPGal for 0–12 h and diluted to a density of 500–1000 cells per plate (SD-URA-LEU). Fourth, yeast were transferred to SD-URA-LEU and SD-HIS plates and grown for 1 day prior to imaging. The number of surviving colonies on the SD-HIS condition was quantified; all were confirmed as diploids.

Numerous safeguards were implemented to ensure contained use of all yeast strains harbouring gene drives. Briefly, these included (i) use of the artificial sequences at the *HIS3* locus, (ii) flanking [u2] sites at the drive cassette itself providing the option for rapid self-excision and removal [40], (iii) placement of the sgRNA expression module on an unstable high-copy (2µ) vector, (iv) poor sporulation of the *S. cerevisiae* BY4741/BY4742 background, (v) constant repression of Cas9 expression until required and (vi) careful inactivation of all yeast strains (cultures, plates and consumables) by autoclaving.

### Fluorescence microscopy, imaging and graphics

Yeasts were grown overnight in a pre-induction culture, back-diluted into medium containing galactose for 4.5 h, washed and prepared on a microscope slide. Cells were imaged using an inverted Leica DMI6500 fluorescence microscope (Leica Microsystems, Buffalo Grove, IL) with a ×100 lens, and fluorescence filters (Semrock, GFP- 4050B-LDKM-ZERO, mCherry-C-LDMK-ZERO). A Leica DFC340 FX camera, Leica Microsystems Application Suite software and ImageJ (National Institute of Health) were used. All images were obtained using identical exposure times and were rescaled together. The ‘merged’ images do not contain any additional processing. Representative cells were chosen for each image. Quantification of the plasma membrane (PM) (maximum) pixel value was done by measuring the cell periphery with a line tool; the cytosol (mean) pixel value was obtained using a line tool. Ten independent measurements were made per cell. Samples were analysed in a single-blind fashion.

Molecular graphics were performed with the University of California, San Francisco Chimera package (Resource for Biocomputing, Visualization and Informatics) [43].

## Results

### Cas9-based editing in haploid yeast

**Fig. 1.**
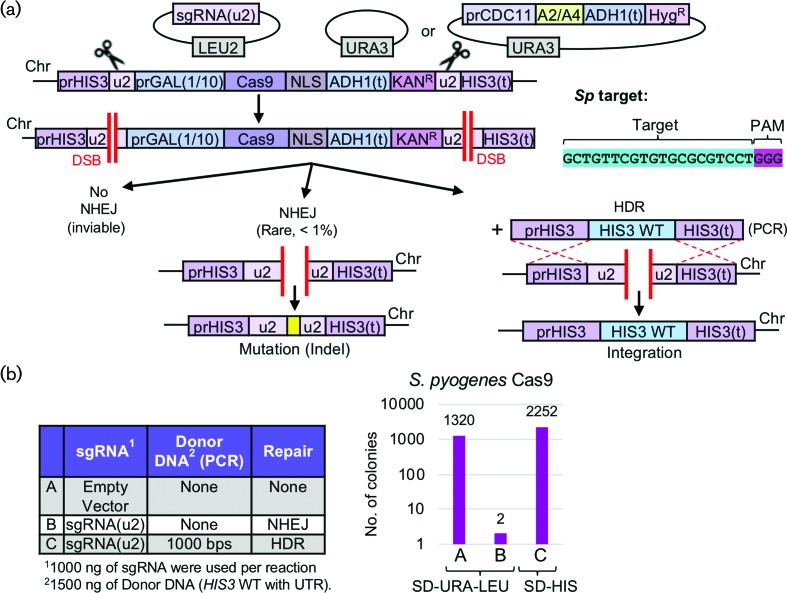
CRISPR/Cas9 editing of a haploid yeast genome using *S. pyogenes* Cas9 and an artificial target system. (a) Schematic of the yeast Cas9 expression platform at the endogenous *HIS3* locus. The Cas9 gene is under control of the inducible *GAL1/10* promoter and the locus is marked with the Kan^R^ cassette. The entire expression module is flanked by two identical artificial [u2] sites (32 inserted base pairs including a PAM sequence), as previously described. A high-copy *LEU2*-marked plasmid harbours the sgRNA[u2] cassette whereas a *URA3*-based plasmid is also present (empty or expressing an anti-CRISPR gene). *S. pyogenes* Cas9 was synthesized with a yeast codon bias and integrated. (b) Yeast strain GFY-2383 harbouring an empty *URA3*-based plasmid (pRS316) was cultured in pre-induction medium (raffinose/sucrose mixture) overnight at 30 °C, back-diluted to an OD_600_ of approximately 0.35 in YPGal medium and grown for 4.5 additional hours. Cells were harvested, transformed with the equimolar amounts of an empty vector control (A, pRS425) or sgRNA[u2] plasmid (B, pGF-V809), recovered overnight in fresh YPGal medium and plated onto SD-URA-LEU selection plates. In one sample, the guide RNA plasmid was co-transformed with a PCR fragment (C, WT *HIS3* ORF with 1000 bp of flanking 5′ and 3′ UTR). The total number of surviving colonies was quantified and graphed on a log_10_ scale.

Given the enormous utility and versatility of the CRISPR/Cas editing system across many fields of scientific inquiry, we have developed a programmable, artificial editing system in budding yeast for use in analysing genome editing (haploids) and gene drives (diploids) [40]. Our system utilizes the presence of artificially programmed sites [36] that contain a maximum mismatch to the yeast genome and are positioned flanking the introduced Cas9 expression cassette and target locus of interest (Fig. 1a). In this way, (i) off-target effects are virtually eliminated, (ii) multiplexing at identical targets requires only a single guide RNA and (iii) gene drive containment and security are maximized. Here, our *in vivo* editing assay was utilized to assess whether *S. pyogenes* Cas9 could efficiently target the yeast genome (Fig. 1). Yeast codon optimized Cas9 (Fig. S1) harbouring a C-terminal NLS signal was integrated under control of an inducible *GAL1/10* promoter and flanked by two identical [u2] sites including the PAM sequence 5′-NGG-3′. Targeting of the dual identical [u2] sites caused full excision of the Cas9 cassette and Kan^R^ marker. Survival of the yeast cell required non-homologous end joining to repair the DSB in the absence of any provided donor DNA. However, given that exact repair of the dual [u2] sites allows for the formation of a new [u2] site (Fig. 1a), subsequent editing will cause cell death unless a sequence modification occurs to disrupt the target site. We have previously demonstrated [36, 40] that successful editing with *S. pyogenes* Cas9 causes cell inviability. In our haploid editing assay, plasmids expressing the sgRNA cassette were transformed into yeast (selecting only for the presence of the plasmid marker). An identical protocol was performed by co-transforming the guide plasmid and a PCR fragment providing donor DNA for homologous recombination (Figs 1b and S2). The total number of surviving colonies demonstrated that editing was very efficient with *S. pyogenes* Cas9.

**Fig. 2.**
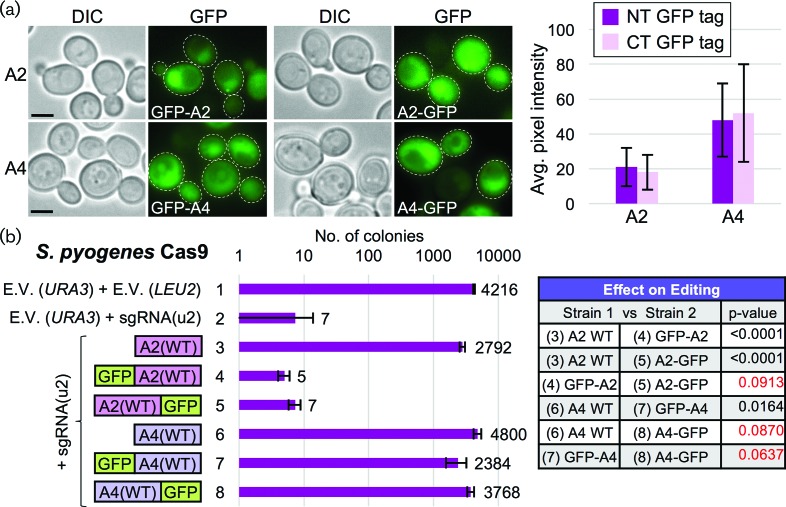
Inhibition of *S. pyogenes* Cas9 editing through *in vivo* expression of AcrIIA2 and AcrIIA4. (a) The anti-CRISPR genes AcrIIA2 and AcrIIA4 were cloned (pGF-IVL1384 to pGF-IVL1387) under control of the *CDC11* promoter on *CEN*-based plasmids, tagged with GFP at either their N- or C-terminus, transformed into WT yeast (BY4741) and imaged by fluorescence microscopy. White dotted outline, cell periphery. Scale bar, 3 µm. Representative cells are illustrated (*left*). An average pixel intensity (cytosol) was measured for individual cells (*right, n*=30–75 cells per genotype). Error, sd. (b) The haploid yeast strain harbouring *S. pyogenes* Cas9 from Fig. 1 (GFY-2383) was first transformed with plasmids expressing either GFP-tagged (a) or untagged (pGF-IVL1336 and pGF-IVL1337) AcrIIA2 and AcrIIA4 constructs. Second, following induction of Cas9 in galactose, yeasts were transformed with equimolar amounts of sgRNA[u2]-containing plasmid (pGF-V809), and the total number of colonies was quantified on SD-URA-LEU plates in triplicate. Error, sd. Select comparisons between experimental conditions (*left*) were analysed using an unpaired *t*-test. Red text highlights *P*-values greater than 0.05.

Our aim was to provide a controlled setting to test for inhibition of Cas9 editing *in vivo* by the newly identified class of anti-CRISPR proteins [23, 24]. These proteins evolved within bacteriophages to counteract the enzymatic function of the Cas9 nuclease. The application of such a naturally occurring counter to the CRISPR editing system could have great potential in many areas of current investigation. We synthesized two anti-CRISPR proteins – AcrIIA2, AcrIIA4 – with a yeast codon bias and expressed them *in vivo* to address whether they could efficiently inhibit the editing function of Cas9. Expression of AcrIIA2 or AcrIIA4 tagged with GFP and under control of the *CDC11* promoter element [44, 45] provided detectible levels of expression within both the cytosol and nuclei of WT yeast cells (Fig. 2a). We observed a higher steady-state level of AcrIIA4 protein in multiple experiments compared to AcrIIA2. GFP-tagged and untagged versions of the AcrIIA2 and AcrIIA4 proteins were expressed in haploid yeast containing *Sp*Cas9 and were transformed with the sgRNA plasmid (Fig. 2b). We found significant inhibition of Cas9 editing in cells expressing either WT AcrIIA2 or AcrIIA4; however, tagging of AcrIIA2 with GFP at either the N- or C-terminus impaired its ability to inhibit editing, distinct from AcrIIA4, which could tolerate the presence of full-length GFP.

**Fig. 3.**
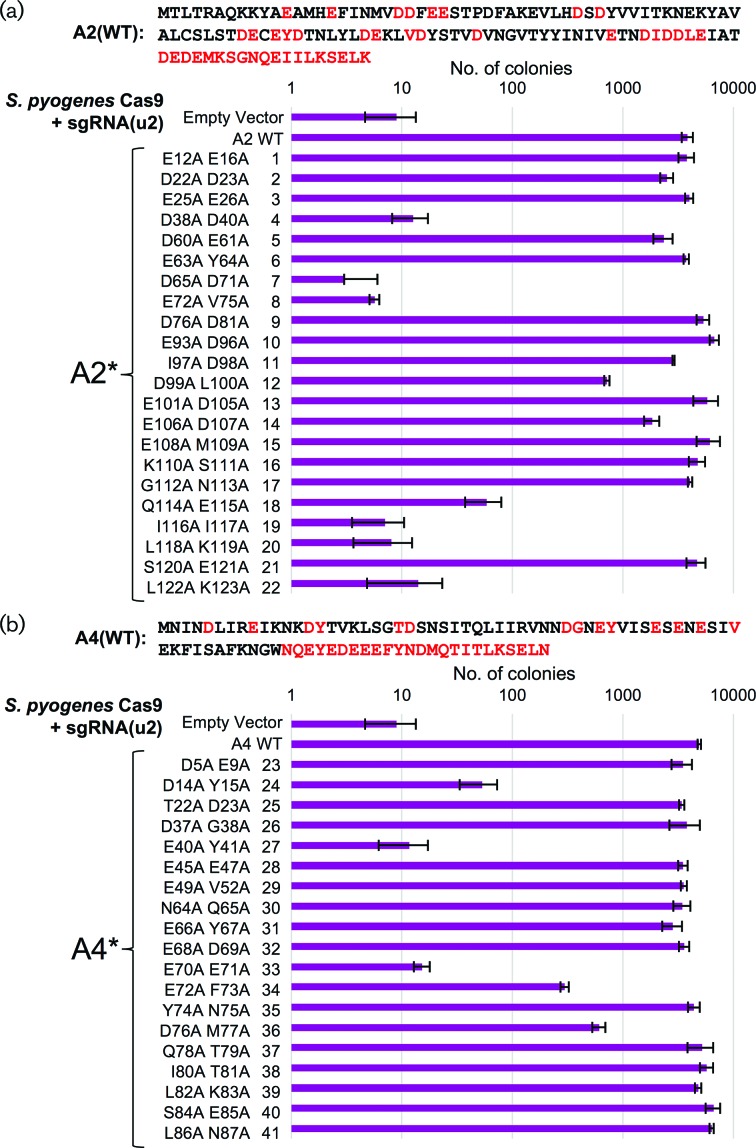
Unbiased alanine scan of the AcrIIA2 and AcrIIA4 proteins and effects on inhibition of *S. pyogenes* Cas9 editing. (a) The AcrIIA2 protein was mutated using pairs of alanine substitutions – red text highlights all amino acids included in the mutation analysis (*top*). Yeast (GFY-2383) were first transformed with all *URA3*-based plasmids: (i) empty pRS316, (ii) WT AcrIIA2 (pGF-IVL1336) or (iii) mutant AcrIIA2 (pGF-V1399 to pGF-V1420). Second, editing of haploid yeast was performed as previously described (see Methods). Briefly, following induction of Cas9, sgRNA[u2] plasmid (pGF-V809) was transformed, recovered overnight and plated to SD-URA-LEU medium. The total number of surviving colonies was quantified in triplicate. Error, sd. (b) A similar mutational analysis was performed on the A4 protein as in (a). Plasmids included: (i) empty pRS316, (ii) WT A4 (pGF-IVL1337) and (iii) mutant A4 (pGF-V1421 to pGF-V1439).

In order to determine which residue(s) or motifs within AcrIIA2 and AcrIIA4 were necessary and/or sufficient for their inhibitory function, we constructed a series of small deletions from either the N- or C-terminus of each protein. However, deletion of only ten residues from either terminus was sufficient to destroy the inhibitory function (Fig. S3). Therefore, we performed an unbiased alanine scan across both anti-CRISPR proteins. Pairs of adjacent residues were chosen as well as several combinations of acidic residues (as both proteins contain many Asp and Glu amino acids). Editing in haploid yeast was performed on all alanine mutants with an identical guide RNA (Fig. 3). For AcrIIA2, 6/22 mutants caused a total loss of inhibitory function, 2/22 mutants caused an intermediate level of inhibition and 14/22 had little to no effect on editing (Fig. 3a). For AcrIIA4, 2/19 mutants caused a loss of inhibition, 3/19 displayed an intermediate range and 14/19 had little to no effect on protein function (Fig. 3b). These data demonstrate that alteration of the AcrIIA2/A4 primary sequence may provide a means to titrate inhibition of *Sp*Cas9 *in vivo*.

### Inhibition of nuclease-based gene drives

**Fig. 4.**
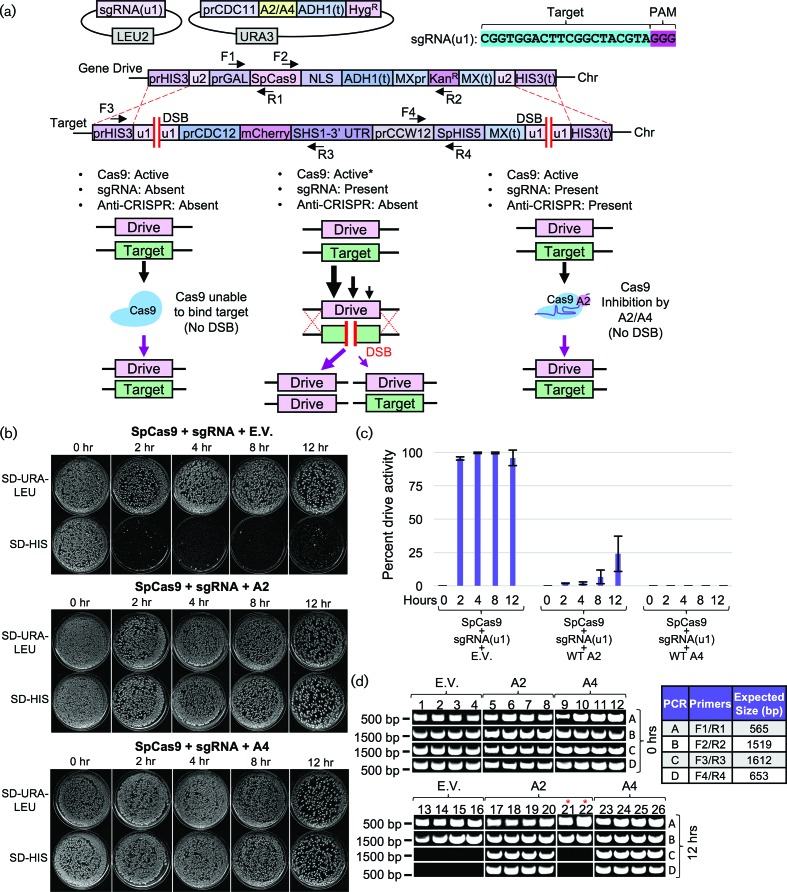
AcrIIA2 and AcrIIA4 can inhibit CRISPR-based gene drives in yeast. (a) Design of an artificial gene drive system in *S. cerevisiae*. The haploid ‘gene drive’ strain (harbouring inducible *S. pyogenes* Cas9) was built in *MATa* yeast (GFY-2383) and included (i) a *LEU2*-based high copy plasmid with the sgRNA[u1] cassette and (ii) the *URA3*-based *CEN* plasmid expressing untagged AcrIIA2 or AcrIIA4 (*top*). Two nearly isogenic ‘target’ strains were built in *MAT****α*** yeast (GFY-3206 and GFY-3207) containing an artificial target gene and selectable *HIS5* marker (from *S. pombe*). The entire construct was flanked with two [u1] artificial target sites. *Middle*, three scenarios are depicted involving (i) a fully active gene drive, (ii) partially active drive activity and (iii) fully inhibited drive activity due to the presence of the anti-CRISPR proteins. (b) Activation of an artificial gene drive system in yeast. First, yeast were transformed with (i) either an empty vector (pRS316) or plasmid expressing AcrIIA2 (pGF-IVL1336) or AcrIIA4 (pGF-IVL1337) and (ii) the sgRNA[u2] plasmid (pGF-V1220), and mated to the target strains (pGF-3206 and pGF-3207) on rich medium (containing dextrose) for 24 h at 30 °C. Second, diploid yeast were obtained by velvet transfer of all colonies to SD-URA-LEU-HIS medium for two consecutive rounds of selection. Third, cultures of pre-induction medium (raffinose/sucrose lacking leucine and lacking uracil) were grown overnight, back-diluted into YPGal and cultured for between 0 and 12 h. Fourth, cells were harvested, washed and diluted to approximately 500–1000 cells per plate (SD-URA-LEU medium) and grown for 2–3 days. Fifth, yeasts were transferred by velvet to an identical plate type and SD-HIS medium for an additional 24 h incubation before imaging. Representative plates for each time point are illustrated. (c) The total number of surviving colonies was quantified for each plate type in duplicate. ‘Gene drive activity’ was illustrated as the proportion of sampled colonies (*n*=100–300 colonies per plate) sensitive to the SD-HIS condition (e.g. 99 % of colonies present on SD-URA-LEU plate but absent on the SD-HIS plate corresponds to 99 % gene drive activity). (d) Molecular analysis of diploid yeast following gene drive activation. Clonal isolates were obtained from the 0 and 12 h time points (SD-URA-LEU plate), chromosomal DNA was purified and PCRs were performed on the diploid genomes. Primer combinations and the expected fragment sizes (*right*) are illustrated in the gene drive schematic (a). Four representative isolates from each genotype are illustrated – for the gene drive containing AcrIIA2, two isolates displaying no growth on SD-HIS were also tested (red asterisk).

One application of Cas9 inhibition that has been previously proposed has been to halt the action and progression of a nuclease-based gene drive. Given the potential for this *super*-Mendelian drive of a genetic element through a population, we tested whether AcrIIA2 and AcrIIA4 could inhibit a gene drive in budding yeast. The design of our gene drive system [40] includes use of flanking [u1] sites, an artificial target gene and a selectable marker (*S. pombe HIS5*) to assess ‘success’ of the drive following activation of Cas9 (Fig. 4a). The plasmids containing AcrIIA2 or AcrIIA4 and the sgRNA plasmid were transformed into yeast prior to activation of Cas9. Following mating, diploid selection and induction of Cas9 (see Methods), we quantified success of the active gene drive within a population of yeast by scoring loss of the genetic marker within the target genome (Fig. 4b, c). An active drive system results in >99 % activity following expression of Cas9. However, inclusion of either AcrIIA2 or AcrIIA4 in the same strain caused a near total loss of gene drive activity (>99.9 % inhibition by AcrIIA4). Characterization of samples of surviving diploids demonstrated that strains expressing the anti-CRISPR proteins maintained copies of both the gene drive and target cassettes whereas diploids following action of the drive only contain two identical copies of the gene drive allele (Fig. 4d).

**Fig. 5.**
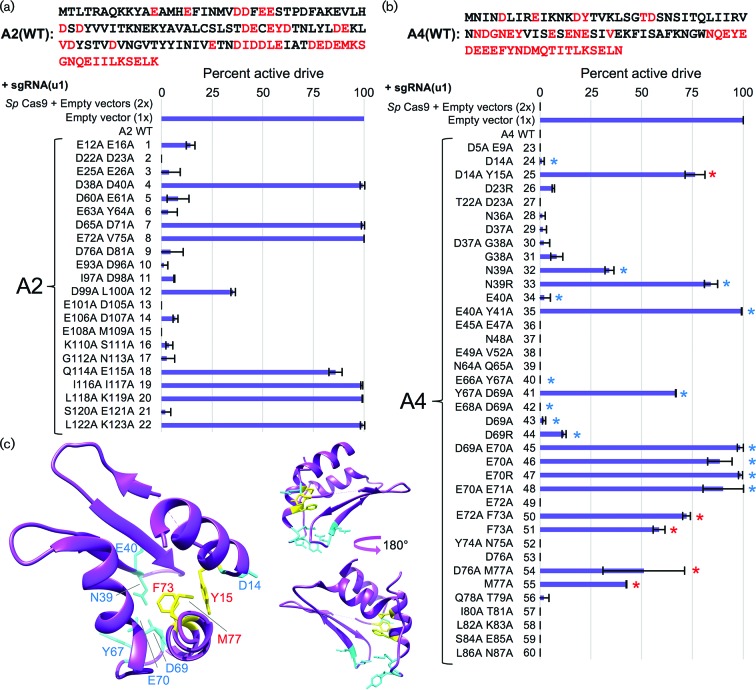
Mutational analysis of the Cas9 inhibitory function of AcrIIA2 in an active gene drive system. (a) Vectors expressing AcrIIA2 mutants (Fig. 3, *top left*, red text) and the sgRNA[u1] plasmid (pGF-V1220) were transformed into yeast along with controls (2x, empty pRS316, pRS425; 1x, empty pRS316, sgRNA[u1] plasmid). Gene drive strains were mated to the target strains (GFY-3206 and GFY-3207) and the diploid strains were induced in rich media containing galactose for 5 h before plating. The percentage of colonies displaying an active drive system was quantified in duplicate. Error, sd. (b) Vectors expressing AcrIIA4 mutants (Fig. 3, *top right*, red text) and the sgRNA[u1] plasmid were analysed as in (a). Additional mutants not previously tested were also included (pGF-V1470 to pGF-V1485 and pGF-V1534 to pGF-V1536). Blue asterisks, residues tested by previous groups [25, 26] shown to contribute to AcrIIA4 association with sgRNA-loaded Cas9. Red asterisks, mutational substitutions displaying a partial loss of inhibitory function not previously documented. (c) *Left,* crystal structure of the AcrIIA4 protein bound to Cas9/sgRNA (PDB 5XBL). Cyan-labelled residues (and side chains) are residues previously demonstrated to be critical for AcrIIA4 association with Cas9. Yellow-labelled residues (red text) illustrate three substitutions found to partially reduce AcrIIA4 inhibitory function *in vivo. Right,* side views of AcrIIA4 with a 180° rotation.

**Fig. 6.**
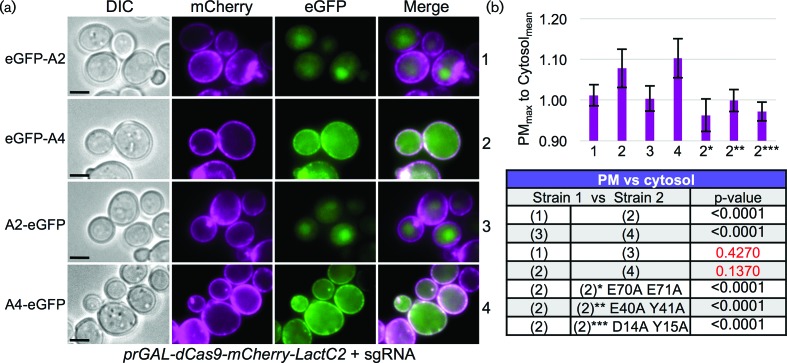
*In vivo* dCas9 association assay for anti-CRISPR proteins. (a) A yeast strain was constructed harbouring an inducible dCas9 (D10A H840A) fused to both mCherry and LactC2 at its C-terminus (GFY-3104) and transformed with (i) a sgRNA[u1] plasmid (pGF-V1220) and (ii) GFP-tagged AcrIIA2/A4-containing plasmids (pGF-IVL1384 to pGF-IVL1387). Strains were cultured overnight in raffinose/sucrose medium lacking uracil and lacking leucine, back-diluted into synthetic medium containing galactose also lacking uracil and leucine, and grown for 4.5 h at 30 °C. Cells were harvested, washed with water and imaged by fluorescence microscopy. Representative images are displayed. Scale bar, 3 µm. (b) A measure of the ratio between the maximum pixel intensity located on the PM was compared to a sampling of the average cytosolic pixel intensity for the GFP signal (*n*=15–30 cells per genotype). Ten random individual measurements were taken for both the PM and cytosolic levels per cell. Error, sd. Three additional AcrIIA4 constructs were tested (asterisks) containing sets of two alanine substitutions (pGF-IVL1431 to pGF-IVL1433). *Bottom*, select strains were compared using an unpaired *t*-test. Red text, *P*-values greater than 0.05.

We also tested the inhibitory function of all AcrIIA2 and AcrIIA4 mutants in the context of this drive system. We observed an inverse correlation between anti-CRISPR inhibitory function and gene drive activity (Figs 3 and 5). Given the recent interest in AcrIIA4 by other groups [25, 26, 34], we also included additional mutations (single and double residue changes) and tested their contribution to Cas9 gene drive inhibition (Fig. 5b, Table S3). Of note, our mutational analysis is consistent with two recent studies that solved the crystal structure of AcrIIA4 bound to sgRNA-loaded *Sp*Cas9 [25, 26]. Both groups highlighted AcrIIA4 residues N39, E40, Y67, D69 and E70 as critical for association with Cas9 by *in vitro* binding and nuclease-dependent DNA cleavage assays. Mutational substitution of these critical residues (and in combination) were shown to reduce or eliminate binding and prevent AcrIIA4 inhibition of Cas9 [25, 26]. Here, we provide *in vivo* evidence that mutation of these residues (to alanine or arginine) largely decreases the effectiveness of the AcrIIA4 as a Cas9/gene drive inhibitor. While the strongest single mutations included N39R and E70R, we and others have demonstrated a further decrease in binding (and function) by combinatorial substitutions (Fig. 5b, Table S3). However, our analysis also revealed that residues Y15, F73 and M77 provided an *intermediate* reduction of inhibition that might be further exploited to titrate the level of Cas9 activity (Fig. 5b). Surprisingly, these three residues are found in close proximity within the AcrIIA4 structure forming part of the hydrophobic interior and do not appear to contact Cas9 (Fig. 5c). For three of the most potent mutant combinations resulting in a loss of inhibition by AcrIIA4 (E70A/E71A, E40A/Y41A and D14A/Y15A), we developed an *in vivo* dCas9 association assay (Fig. 6). Expression of a membrane-tethered (via a Lact-C2 domain and lacking any NLS sequence), mCherry-tagged dCas9 primed with a (nonsense) sgRNA was co-expressed with WT AcrIIA2 or AcrIIA4 in yeast. We observed recruitment of the GFP-tagged AcrIIA4 protein to the PM – this co-localization was dependent on (i) the presence of dCas9 (Fig. S4) and (ii) a fully functional AcrIIA4 protein as all three alanine mutants failed to localize to the PM (Fig. 6b).

**Fig. 7.**
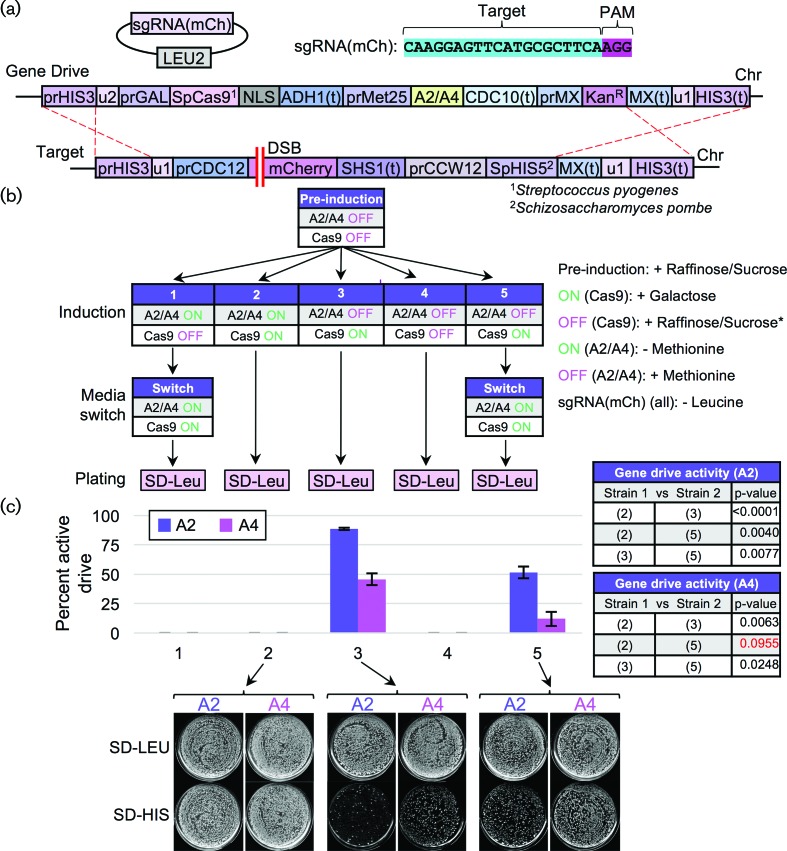
Temporal control of anti-CRISPR expression can modulate the overall activity of a nuclease-based gene drive. (a) Schematic of the gene drive system harbouring an inducible AcrIIA2 or AcrIIA4 inhibitor within the cassette. Modifications to the previously tested gene drive (Figs 4 and 5) included integration of a second inducible expression cassette (*MET25* promoter) proximal to the *S. pyogenes* gene drive system controlling expression of either AcrIIA2 or AcrIIA4. The sgRNA targeted the mCherry gene within the target strains. (b) The two gene drive strains (GFY-3285 and GFY-3287) were transformed with the sgRNA(mCh) plasmid (pGF-425+IVL1277), mated to the target strains (GFY-3206 and GFY-3207) and diploids were selected on SD-LEU-HIS plates (three consecutive times). Diploids were then cultured overnight in pre-induction medium (raffinose/sucrose lacking leucine and containing methionine). Five distinct growth conditions were tested (labelled 1–5) altering the order of either Cas9 induction, AcrIIA2/A4 induction or control conditions. Cas9 induction included culturing in medium containing galactose. Inhibition (asterisk) of Cas9 expression included use of the raffinose/sucrose mixture. Activation of AcrIIA2/A4 included culturing in medium lacking methionine (repression by addition of methionine). All culturing steps also lacked leucine to maintain the sgRNA(mCh) plasmid. For conditions (1) and (5), a media switch occurred – after 2 h, yeast were harvested, washed four times with water and resuspended in the new media type for an additional 2 h. All diploids were plated on SD-LEU medium as previously described (500–1000 cells per plate). (c) The percentage of drive activity was quantified in duplicate. Error, sd. *Below*, representative plates from selected conditions are illustrated. *Right*, select combinations of experimental conditions for AcrIIA2 and AcrIIA4 were compared using an unpaired *t*-test. Red text, *P*-value of greater than 0.05.

Finally, we developed a gene drive system harbouring an inducible AcrIIA2/A4 within the drive cassette (Fig. 7a). While *Sp*Cas9 expression was activated in the presence of galactose, AcrIIA2/A4 expression was induced by lack of methionine (*MET25* promoter). Several experimental conditions were tested including co-expression of both Cas9 and the inhibitor, expression of only one component prior to the other and relevant controls (Fig. 7b). A recent study also provided data that inhibition of Cas9 editing was sensitive to the timing of delivery/presence of the inhibitor protein [34]. Here, we observed that drive inhibition can be titrated to various levels of activity (including full inhibition) based on (i) the choice of either AcrIIA2 or AcrIIA4 and (ii) the timing of anti-CRISPR expression. While the *MET25* promoter does allow for some leaked transcript of AcrIIA2/A4 (Fig. 7c, *condition 3*), our design demonstrates that a gene drive could be programmed to halt or titrate drive activity depending on the intended application and choice of inducible promoter systems.

## Discussion

Our study has demonstrated that the anti-CRISPR proteins AcrIIA2 and AcrIIA4 are able to inhibit the function of *S. pyogenes* Cas9 *in vivo* within haploid yeast and an active gene drive system. We found that epitopes or gene fusions were not tolerated on either terminus of AcrIIA2 in contrast to AcrIIA4. Therefore, we performed an extensive mutational scan across both proteins to (i) determine which residues were necessary for function and (ii) determine whether specific substitutions might provide an intermediate level of Cas9 inhibition. Based on the recent AcrIIA4 crystal structure [25, 26], it is evident that inhibition of *Sp*Cas9 is through mimic of the PAM-binding motif within the nuclease. Several critical residues on AcrIIA4 were shown to be necessary for binding of Cas9 and inhibition of function *in vitro.* Our approach has confirmed these findings, revealed additional residues necessary for function within AcrIIA2/A4, and identified several positions that provided a *partial* loss of function. Use of a tagged or mutated AcrIIA4 protein coupled with temporal control over expression (as demonstrated with an inducible promoter) could provide an expanded suite of options for control of Cas9 function *in vivo*, and especially as a potent gene drive inhibitor.

The anti-CRISPR proteins provide several advantages over other (current or proposed) methods of Cas9 inhibition. First, it is a separate peptide not requiring translational fusion to Cas9/dCas9 – this includes the ability to control timing and level of expression of the inhibitor separate from the nuclease. We suspect that regulation of subcellular location (of both Cas9 and/or the inhibitor) may provide even more options to enhance or restrict interaction with the Cas9/guide RNA complex. Second, this class of proteins is extremely small (87 residues for AcrIIA4) and, in some cases, can tolerate tags (GFP) three times their size and still function to inhibit Cas9. Third, inhibition appears to be titratable based on the amount of AcrII protein present and the inclusion of specific amino acid substitutions. Fourth, these proteins have evolved as a natural DNA mimic and may inform further design and/or optimization of new classes of peptides or small molecules. Our study has provided the first documented use of the anti-CRISPR proteins AcrIIA2 and AcrIIA4 to inhibit a gene drive system. Future nuclease-based gene drives could include an inducible drive inhibitor within the original cassette to provide a useful off-switch for the system, control the timing of drive activation or halt propagation of a current drive element while a second (‘anti-drive’) drive replaces or destroys the first.

## Supplementary Data

743 Supplementary File 1
