# Corrigendum: Gene drive inhibition by the anti-CRISPR proteins AcrIIA2 and AcrIIA4 in *Saccharomyces cerevisiae*

**DOI:** 10.1099/mic.0.000678

**Published:** 2018-05-29

**Authors:** Erianna M. Basgall, Samantha C. Goetting, Megan E. Goeckel, Rachael M. Giersch, Emily Roggenkamp, Madison N. Schrock, Megan Halloran, Gregory C. Finnigan

**Affiliations:** ^1^​ Department of Biochemistry and Molecular Biophysics, 141 Chalmers Hall, Kansas State University, Manhattan, KS 66506, USA; ^2^​ Department of Biology, 116 Ackert Hall, Kansas State University, Manhattan, KS 66506, USA

**Keywords:** biotechnology, anti-CRISPR, CRISPR, regulating gene drives, gene drive, Cas9, sgRNA

There was an error in the Conflicts of interest statement in the published article. It should state:

‘G. C. F. (Kansas State Univ.) has filed for a provisional patent on September 29, 2017 (U.S. Provisional Patent Application Serial No. 62/565,651, “Programmed modulation of CRISPR/Cas9 activity”) followed by a full patent filing on January 31, 2018 regarding the technology presented in this work.’

The author apologizes for any inconvenience caused.

