# Supplementary material for: Gene drive inhibition by the anti-CRISPR proteins AcrIIA2 and AcrIIA4 in Saccharomyces cerevisiae
Source: Microbiology (Reading). 2018 Feb 28;164(4):464–74. doi: 10.1099/mic.0.000635 (PMC5982135; doi:10.1099/mic.0.000635)
Supplement: Supplementary File 1 [file mic-164-464-s001.pdf]

SUPPORTING INFORMATION

For

Gene drive inhibition by the anti-CRISPR proteins AcrIIA2 and AcrIIA4 in *Saccharomyces cerevisiae*

Erianna M. Basgall[1†], Samantha C. Goetting[1†], Megan E. Goeckel[1†], Rachael M. Giersch[2], Emily Roggenkamp[1], Madison N. Schrock[1], Megan Halloran[1], and Gregory C. Finnigan[1*]

[1]Department of Biochemistry and Molecular Biophysics, 141 Chalmers Hall, Kansas State University, Manhattan, KS 66506 USA

[2]Department of Biology, 116 Ackert Hall, Kansas State University, Manhattan, KS 66506 USA

[†]Authors contributed equally

*Correspondence to: Gregory C. Finnigan, Phone: (785) 532-6939; FAX; (785) 532-7278; E-mail: gfinnigan@ksu.edu

Figure S1. Cas9 and sgRNA expression cassette sequences used in this study.

S. pyogenes Cas9 (yeast codon bias): Red highlights, START, STOP codons. Purple text, SV40 NLS sequence.

ATGGATAAGAAATACTCTATCGGTTTGGATATTGGTACAAATTCAGTTGGTTGGGCAGTTATTACTGATGAATACAA
GGTTCCATCTAAAAAGTTTAAAGTTTTGGGTAACACTGATAGACATTCTATTAAGAAAAATTTGATTGGTGCTTTGT
TATTTGATTCTGGTGAAACTGCTGAAGCAACAAGATTGAAAAGAACTGCAAGAAGAAGATACACAAGAAGAAAGAAT
AGAATCTGTTATTTGCAAGAAATTTTCTCTAACGAAATGGCTAAGGTTGATGATTCTTTCTTTCATAGATTGGAAGA
ATCATTTTTAGTTGAAGAAGATAAGAAACATGAAAGACATCCAATCTTCGGTAACATCGTTGATGAAGTTGCTTACC
ATGAAAAGTACCCAACAATCTATCATTTGAGAAAGAAATTGGTTGATTCAACTGATAAGGCAGATTTGAGATTGATA
TATTTGGCTTTAGCACATATGATCAAGTTTAGAGGTCATTTCTTGATCGAGGGTGACTTGAATCCAGATAATTCTGA
TGTTGATAAGTTGTTTATTCAATTAGTTCAAACATATAATCAATTGTTTGAAGAAAATCCAATTAATGCTTCTGGTG
TTGATGCTAAGGCAATCTTGTCAGCAAGATTGTCTAAGTCAAGAAGATTGGAAAATTTGATCGCTCAATTACCAGGT
GAAAAGAAAAATGGTTTGTTCGGTAATTTGATCGCATTGTCTTTGGGTTTGACACCAAACTTCAAGTCAAACTTCGA
TTTGGCTGAAGATGCAAAGTTGCAATTGTCTAAGGATACTTACGATGATGATTTGGATAATTTGTTGGCTCAAATTG
GTGACCAATATGCAGATTTGTTTTTGGCTGCTAAAAATTTGTCTGATGCTATCTTGTTGTCAGATATCTTGAGAGTT
AACACTGAAATCACAAAGGCTCCATTGTCTGCATCAATGATCAAGAGATACGATGAACATCATCAAGATTTGACTTT
GTTGAAGGCATTGGTTAGACAACAATTACCAGAAAAGTACAAGGAAATTTTCTTTGATCAATCTAAAAATGGTTATG
CTGGTTACATTGATGGTGGTGCATCTCAAGAAGAATTCTACAAGTTTATTAAGCCAATCTTGGAAAAGATGGATGGT
ACAGAAGAATTGTTAGTTAAATTGAACAGAGAAGATTTGTTAAGAAAACAAAGAACTTTCGATAACGGTTCTATCCC
ACATCAAATCCATTTGGGTGAATTACATGCTATCTTGAGAAGACAAGAAGATTTCTACCCATTTTTAAAGGATAACA
GAGAAAAGATTGAAAAGATTTTGACTTTTAGAATTCCATATTACGTTGGTCCATTAGCTCGTGGTAATTCTAGATTT
GCATGGATGACTAGAAAGTCAGAAGAAACTATCACACCATGGAATTTTGAAGAAGTTGTTGATAAAGGTGCTTCTGC
ACAATCTTTTATTGAAAGAATGACAAACTTCGATAAAAATTTGCCAAACGAAAAGGTTTTGCCAAAGCATTCATTGT
TATATGAATACTTTACTGTTTACAATGAATTGACAAAAGTTAAATATGTTACTGAGGGTATGAGAAAACCAGCATTT
TTGTCTGGTGAACAAAAGAAAGCAATCGTTGATTTGTTGTTTAAAACTAACAGAAAGGTTACAGTTAAACAATTGAA
AGAAGATTACTTTAAGAAAATTGAATGTTTTGATTCTGTTGAAATTTCAGGTGTTGAAGATAGATTCAATGCTTCAT
TAGGTACTTACCATGATTTGTTGAAGATTATTAAGGATAAAGATTTCTTGGATAATGAAGAAAATGAAGATATTTTA
GAAGATATTGTTTTAACTTTGACATTATTTGAAGATAGAGAAATGATCGAAGAAAGATTGAAGACATACGCTCATTT
GTTCGATGATAAAGTTATGAAGCAATTGAAGAGAAGAAGATACACTGGTTGGGGTAGATTGTCTAGAAAGTTGATTA
ATGGTATCAGAGATAAGCAATCTGGTAAAACAATCTTGGATTTCTTGAAGTCAGATGGTTTCGCAAACAGAAACTTC
ATGCAATTGATTCATGATGATTCATTGACTTTTAAAGAAGATATCCAAAAAGCTCAAGTTTCTGGTCAGGGTGACTC
ATTGCATGAACATATTGCTAATTTGGCAGGTTCTCCAGCTATTAAGAAAGGTATCTTGCAAACAGTTAAGGTTGTTG
ATGAATTAGTTAAAGTTATGGGTAGACATAAGCCAGAAAACATCGTTATCGAAATGGCTAGAGAAAACCAAACTACA
CAAAAGGGTCAAAAGAATTCAAGAGAAAGAATGAAGAGAATCGAAGAAGGTATTAAAGAATTGGGTTCTCAAATCTT
GAAGGAACATCCAGTTGAAAACACTCAATTGCAAAACGAAAAGTTGTACTTATACTACTTACAAAACGGTAGAGATA
TGTACGTTGATCAAGAATTAGATATCAACAGATTGTCAGATTACGATGTTGATCATATCGTTCCACAATCATTTTTG
AAGGATGATTCAATCGATAATAAGGTTTTGACAAGATCTGATAAGAACCGTGGTAAATCTGATAATGTTCCATCAGA
AGAAGTTGTTAAGAAAATGAAGAACTACTGGAGACAATTGTTAAATGCTAAGTTGATCACTCAAAGAAAGTTCGATA
ATTTGACAAAAGCTGAAAGAGGTGGTTTGTCAGAATTAGATAAAGCAGGTTTTATTAAGAGACAATTAGTTGAAACT
AGACAAATCACAAAGCATGTTGCACAAATCTTGGATTCTAGAATGAACACTAAATATGATGAAAATGATAAATTAAT
TAGAGAAGTTAAAGTTATTACATTAAAATCTAAATTGGTTTCAGATTTTAGAAAAGATTTTCAATTCTACAAAGTTA
GAGAAATTAATAACTATCATCATGCTCATGATGCATACTTGAATGCTGTTGTTGGTACTGCATTGATTAAGAAATAC
CCAAAGTTGGAATCTGAATTCGTTTACGGTGACTACAAGGTTTACGATGTTAGAAAGATGATCGCTAAGTCAGAACA
AGAAATCGGTAAAGCTACAGCAAAGTATTTCTTTTATTCTAACATCATGAATTTCTTTAAAACTGAAATTACATTAG
CTAACGGTGAAATCAGAAAAAGACCATTGATCGAAACTAATGGTGAAACAGGTGAAATTGTTTGGGATAAAGGTAGA
GATTTCGCAACTGTTAGAAAGGTTTTGTCAATGCCACAAGTTAACATCGTTAAGAAAACTGAAGTTCAAACAGGTGG
TTTTTCTAAGGAATCAATCTTGCCAAAGAGAAACTCTGATAAGTTGATTGCTAGAAAGAAAGATTGGGATCCAAAGA
AATATGGTGGTTTTGATTCTCCAACTGTTGCTTACTCAGTTTTAGTTGTTGCAAAGGTTGAAAAGGGTAAATCTAAG
AAATTGAAATCAGTTAAAGAATTGTTAGGTATCACAATCATGGAAAGATCTTCATTCGAAAAGAATCCAATCGATTT
CTTGGAAGCAAAGGGTTACAAGGAAGTTAAGAAAGATTTGATTATTAAGTTGCCAAAGTACTCTTTGTTCGAATTAG
AAAACGGTAGAAAAAGAATGTTAGCTTCAGCTGGTGAATTGCAAAAGGGTAATGAATTGGCTTTGCCATCTAAGTAC
GTTAATTTCTTGTATTTGGCATCTCATTACGAAAAGTTGAAGGGTTCACCAGAAGATAATGAACAAAAACAATTGTT
CGTTGAACAACATAAGCATTATTTGGATGAAATTATTGAACAAATTTCTGAATTTTCAAAAAGAGTTATTTTGGCTG
ATGCAAATTTGGATAAGGTTTTGTCTGCTTACAATAAGCATAGAGATAAGCCAATCAGAGAACAAGCAGAAAACATC

ATCCATTTGTTTACTTTGACAAATTTGGGTGCTCCAGCTGCTTTTAAATACTTCGATACTACAATCGATAGAAAAAG
ATACACTTCTACAAAGGAAGTTTTGGATGCAACATTGATCCATCAATCAATCACTGGTTTGTATGAAACAAGAATTG
ATTTGTCTCAATTGGGTGGTGAC**TCTAGGGCAGACCCAAAGAAAAAGAGGAAAGTATAA**

S. pyogenes sgRNA cassette [u2]: Orange (bold) text, *SNR52* promoter sequence; Blue highlighted text, crRNA guide sequence; Pale blue text, tracrRNA; Green (bold) text, *SUP4* terminator sequence; Black (bold, underlined), flanking restriction sites.

GGATCCTCACTAAAGGGAACAAAAGCTGGAGCT**TCTTTGAAAAGATAATGTATGATTATGCTTTCACTCATATTTAT
ACAGAAACTTGATGTTTTCTTTCGAGTATATACAAGGTGATTACATGTACGTTTGAAGTACAACTCTAGATTTTGTA
GTGCCCTCTTGGGCTAGCGGTAAAGGTGCGCATTTTTTCACACCCTACAATGTTCTGTTCAAAAGATTTTGGTCAAA
CGCTGTAGAAGTGAAAGTTGGTGCGCATGTTTCGGCGTTCGAAACTTCTCCGCAGTGAAAGATAAATGATCGCTGTT
CGTGTGCGCGTCCT**GTTTTAGAGCTAGAAATAGCAAGTTAAAATAAGGCTAGTCCGTTATCAACTTGAAAAAGTGGC
ACCGAGTCGGTGGTGC**TTTTTTTGTTTTTTATGTCT**TCGAGTCATGTAATTAGTTATGTCACGC**CTCGAG**

S. pyogenes sgRNA cassette [u1]:

GGATCCTCACTAAAGGGAACAAAAGCTGGAGCT**TCTTTGAAAAGATAATGTATGATTATGCTTTCACTCATATTTAT
ACAGAAACTTGATGTTTTCTTTCGAGTATATACAAGGTGATTACATGTACGTTTGAAGTACAACTCTAGATTTTGTA
GTGCCCTCTTGGGCTAGCGGTAAAGGTGCGCATTTTTTCACACCCTACAATGTTCTGTTCAAAAGATTTTGGTCAAA
CGCTGTAGAAGTGAAAGTTGGTGCGCATGTTTCGGCGTTCGAAACTTCTCCGCAGTGAAAGATAAATGATCCGGTGG
ACTTCGGCTACGTA**GTTTTAGAGCTAGAAATAGCAAGTTAAAATAAGGCTAGTCCGTTATCAACTTGAAAAAGTGGC
ACCGAGTCGGTGGTGC**TTTTTTTGTTTTTTATGTCT**TCGAGTCATGTAATTAGTTATGTCACGC**CTCGAG**

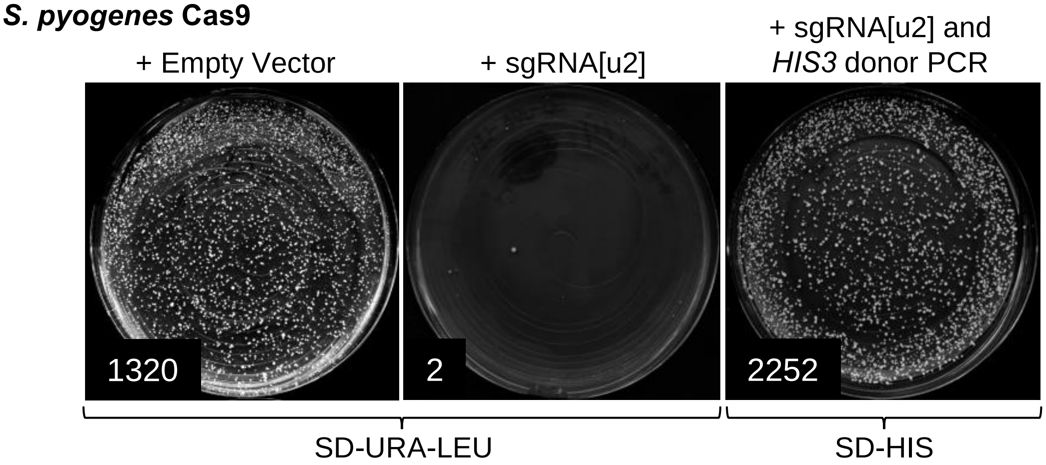

Figure S2. Example editing plates (*S. pyogenes Cas9*) from Fig. 1. A sample SD-URA-LEU (repair via NHEJ) or SD-HIS (repair via HDR) plate are illustrated after 3-5 days of incubation at 30°C. Empty Vector, pRS425. Plasmid harboring sgRNA[u2], pGF-V809. *HIS3* PCR includes approximately 1,000 bp of 5' and 3' UTR.

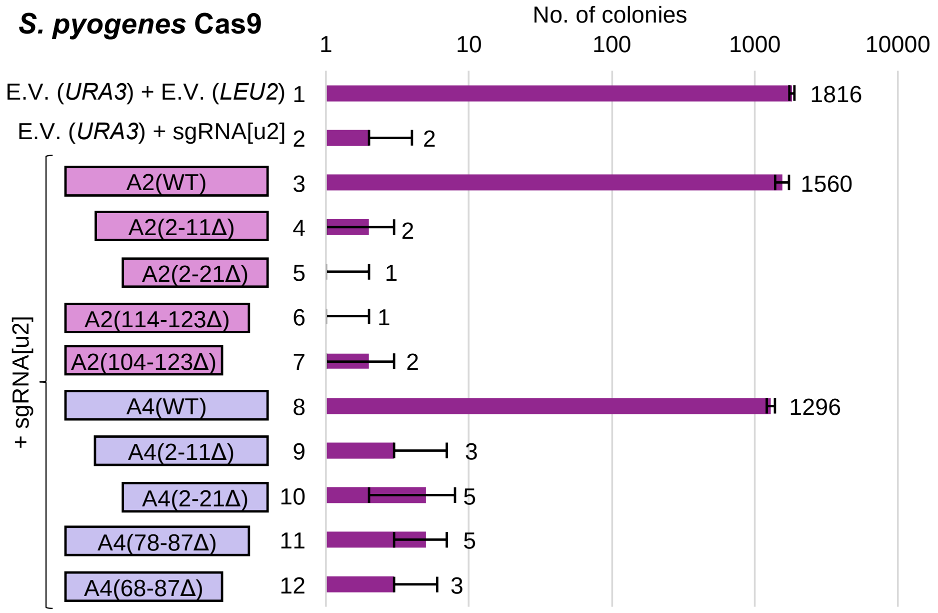

Figure S3. Small deletions of the AcrIIA2 and AcrIIA4 proteins are not tolerated *in vivo* and disrupt the ability to inhibit *S. pyogenes* Cas9 editing. 10 or 20 residues were removed from the A2 and A4 N- or C-termini and expressed *in vivo* on a plasmid under the *CDC11* promoter (pGF-IVL1388 to pGF-IVL1395). Vectors were transformed into yeast harboring an inducible *S. pyogenes* Cas9 expression cassette (GFY-2383). Editing in haploid yeast was performed using the sgRNA[u2] vector (pGF-V809). Yeast were plated onto SD-URA-LEU media and the total number of surviving cells were quantified in triplicate. Error, SD.

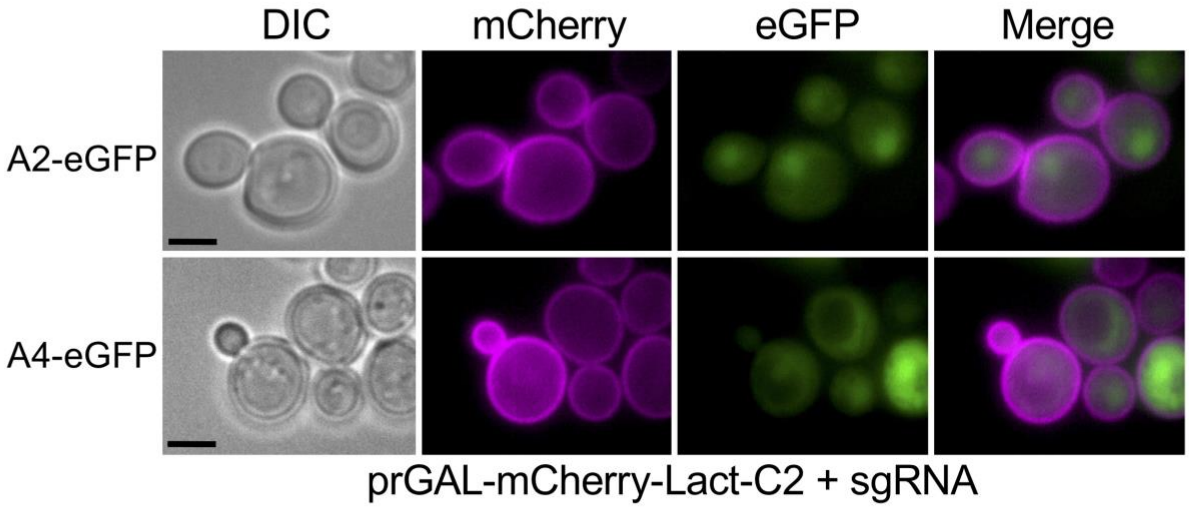

Figure S4. GFP-tagged AcrIIA2 and AcrIIA4 proteins are not recruited to the plasma membrane by mCherry or the LactC2 domain. A strain (GFY-3268) was created which included an mCherry-LactC2 fusion under control of the *GAL1/10* promoter at the *HIS3* locus. Plasmids expressing (i) C-terminally tagged AcrIIA2 or AcrIIA4 (pGF-IVL1386 and pGF-IVL1387) and (ii) sgRNA[u1] (pGF-IVL1220) were transformed into yeast. Cultures were pre-induced overnight in raffinose/sucrose lacking uracil and leucine, back-diluted to medium containing galactose and lacking uracil and leucine, and were grown for 4.5 hr at 30°C. Cells were harvested, washed with water, and imaged by fluorescence microscopy. Representative images are shown. Scale bar, 3 μm.

Table S1. Yeast strains used in this study.

**Strain**	**Genotype**	**Reference**
BY4741	*MAT***a** *his3Δ1 leu2Δ0 met15Δ0 ura3Δ0*	[1]
BY4742	*MAT*α *his3Δ1 leu2Δ0 lys2Δ0 ura3Δ0*	[1]
GFY-2383[a]	BY4741; *his3Δ::(u2)::prGAL1/10::SpCas9::NLS::ADH1(t)::Kan*^{R}*::(u2)::HIS3(t)*	This study
GFY-3206[b]	BY4742; *his3Δ::(u1)::prCDC12::mCherry::NLS::SHS1(t)::prCCW12::SpHIS5::MX(t)::(u1)::HIS3(t)*	This study
GFY-3207[b]	BY4742; *his3Δ::(u1)::prCDC12::mCherry::SHS1(t)::prCCW12::SpHIS5::MX(t)::(u1)::HIS3(t)*	This study
GFY-3285[c]	BY4741; *his3Δ::(u2)::prGAL1/10::SpCas9::NLS::ADH1(t)::prMET25::AcrIIA2::CDC10(t)::prMX::Kan*^{R}*::MX(t)::(u1)::HIS3(t)*	This study
GFY-3287[c]	BY4741; *his3Δ::(u2)::prGAL1/10::SpCas9::NLS::ADH1(t)::prMET25::AcrIIA4::CDC10(t)::prMX::Kan*^{R}*::MX(t)::(u1)::HIS3(t)*	This study
GFY-3104[d]	BY4741; *his3Δ::(u2)::prGAL1/10::dCas9(D10A H840A)::mCherry::LactC2(1-158)::ADH1(t)::Kan*^{R}*::(u2)::HIS3(t)*	This study
GFY-3268[d]	BY4741; *his3Δ::(u2)::prGAL1/10::mCherry::LactC2(1-158)::ADH1(t)::Kan*^{R}*::(u2)::HIS3(t)*	This study

[a]Construction of GFY-2383 was performed by generation of a plasmid (pGF-IVL1192) containing the entire cassette including flanking *HIS3* UTR. The *Streptococcus pyogenes* Cas9 gene was synthesized *de novo* with a yeast codon bias. Two overlapping (120 bp within the Cas9 ORF) PCRs were amplified, digested with *DpnI*, transformed into WT BY4741 yeast, and selected on media containing G418. The artificial site [u2] was placed directly upstream of the *GAL1/10* promoter sequence (814 bp) and downstream of the *MX(t)* sequence [2]. For GFY-2586 and GFY-2583, the [u2] sequence included the *Sp* Cas9 target site and PAM: 5' **GCTGTTCGTGTGCGCGTCCT<u>GGG</u>** 3' (red underlined text is the PAM NGG). A C-terminal NLS (SRADPKKKRKV) was also included [3].
[b]Target yeast strains (GFY-3206 and GFY-3207) were constructed with flanking (u1) sites with the sequence: 5' ATGA**CGGTGGACTTCGGCTACGTA<u>GGG</u>**CGATT 3'. 992 bp of the constitutive *CCW12* promoter were used to drive *S. pombe HIS5* expression.
[c]For strains GFY-3285 and GFY-3287, the following methodology was employed. First, plasmids harboring *ADH1(t)::prMET25::AcrIIA2::CDC10(t)::prCCW12::SpHIS5::MX(t)* (pGF-IVL1410) or the same construct with AcrIIA4 (pGF-IVL1411) were generated [4], PCR amplified in overlapping fragments, *DpnI* treated, integrated into GFY-2383, and colonies were selected for survival on SD-HIS medium and sensitivity to G418. 384 bp of the *MET25* promoter were used. Second, the *HIS5* marker was replaced with the Kan^R MX-based cassette using an amplified fragment containing *CDC10(t)::prMX::*Kan^R*::MX(t)* (from pGF-IVL1412). There is a (u1) site downstream of the *MX(t)* sequence rather than a (u2) site.
[d]Construction of GFY-3104 and GFY-3268 included use of enzymatically dead Cas9 (D10A H840A). A modified site directed mutagenesis protocol [5] was used in introduce substitutions to the ORF in a pUC57 vector prior to *in vivo* plasmid assembly. The LactC2 [6, 7] domain was amplified from pGF-IVL687. The constructs were integrated into the yeast genome as previously described.

Table S2. Plasmids used in this study.

Plasmid	Description	Reference
pRS425	*2μ, LEU2*	[8]
pRS316	*CEN URA3*	[9]
pGF-V809[a]	pRS425; *prSNR52::Sp-sgRNA(u2-20 WT)::SUP4(t)*	This study
pGF-IVL1336[b]	pRS316; *prCDC11::AcrIIA2::ADH1(t)::Hyg*^{R}	This study
pGF- IVL1337[b]	pRS316; *prCDC11::AcrIIA4::ADH1(t)::Hyg*^{R}	This study
pGF-IVL1384[c]	pRS316; *prCDC11::GFP::AcrIIA2::ADH1(t)::Hyg*^{R}	This study
pGF-IVL1385[c]	pRS316; *prCDC11::GFP::AcrIIA4::ADH1(t)::Hyg*^{R}	This study
pGF-IVL1386[d]	pRS316; *prCDC11::AcrIIA2::eGFP::ADH1(t)::Hyg*^{R}	This study
pGF IVL1387[d]	pRS316; *prCDC11::AcrIIA4::eGFP::ADH1(t)::Hyg*^{R}	This study
pGF-V1399[e]	pRS316; *prCDC11::AcrIIA2(L122A K123A)::ADH1(t)*	This study
pGF-V1400	pRS316; *prCDC11::AcrIIA2(S120A E121A)::ADH1(t)*	This study
pGF-V1401	pRS316; *prCDC11::AcrIIA2(L118A K119A)::ADH1(t)*	This study
pGF-V1402	pRS316; *prCDC11::AcrIIA2(I116A I117A)::ADH1(t)*	This study
pGF-V1403	pRS316; *prCDC11::AcrIIA2(Q114A E115A)::ADH1(t)*	This study
pGF-V1404	pRS316; *prCDC11::AcrIIA2(G112A N113A)::ADH1(t)*	This study
pGF-V1405	pRS316; *prCDC11::AcrIIA2(K110A S111A)::ADH1(t)*	This study
pGF-V1406	pRS316; *prCDC11::AcrIIA2(E108A M109A)::ADH1(t)*	This study
pGF-V1407	pRS316; *prCDC11::AcrIIA2(E106A D107A)::ADH1(t)*	This study
pGF-V1408	pRS316; *prCDC11::AcrIIA2(E101A D105A)::ADH1(t)*	This study
pGF-V1409	pRS316; *prCDC11::AcrIIA2(D99A L100A)::ADH1(t)*	This study

pGF-V1410	pRS316; *prCDC11::AcrIIA2(I97A D98A)::ADH1(t)*	This study
pGF-V1411	pRS316; *prCDC11::AcrIIA2(E72A V75A)::ADH1(t)*	This study
pGF-V1412	pRS316; *prCDC11::AcrIIA2(D65A D71A)::ADH1(t)*	This study
pGF-V1413	pRS316; *prCDC11::AcrIIA2(E63A Y64A)::ADH1(t)*	This study
pGF-V1414	pRS316; *prCDC11::AcrIIA2(D60A E61A)::ADH1(t)*	This study
pGF-V1415	pRS316; *prCDC11::AcrIIA2(E93A D96A)::ADH1(t)*	This study
pGF-V1416	pRS316; *prCDC11::AcrIIA2(D76A D81A)::ADH1(t)*	This study
pGF-V1417	pRS316; *prCDC11::AcrIIA2(D38A D40A)::ADH1(t)*	This study
pGF-V1418	pRS316; *prCDC11::AcrIIA2(E25A E26A)::ADH1(t)*	This study
pGF-V1419	pRS316; *prCDC11::AcrIIA2(D22A D23A)::ADH1(t)*	This study
pGF-V1420	pRS316; *prCDC11::AcrIIA2(E12A E16A)::ADH1(t)*	This study
pGF-V1421	pRS316; *prCDC11::AcrIIA4(L86A N87A)::ADH1(t)*	This study
pGF-V1422	pRS316; *prCDC11::AcrIIA4(S84A E85A)::ADH1(t)*	This study
pGF-V1423	pRS316; *prCDC11::AcrIIA4(L82A K83A)::ADH1(t)*	This study
pGF-V1424	pRS316; *prCDC11::AcrIIA4(I80A T81A)::ADH1(t)*	This study
pGF-V1425	pRS316; *prCDC11::AcrIIA4(Q78A T79A)::ADH1(t)*	This study
pGF-V1426	pRS316; *prCDC11::AcrIIA4(D76A M77A)::ADH1(t)*	This study
pGF-V1427	pRS316; *prCDC11::AcrIIA4(Y74A N75A)::ADH1(t)*	This study
pGF-V1428	pRS316; *prCDC11::AcrIIA4(E72A F73A)::ADH1(t)*	This study
pGF-V1429	pRS316; *prCDC11::AcrIIA4(E70A E71A)::ADH1(t)*	This study
pGF-V1430	pRS316; *prCDC11::AcrIIA4(E68A D69A)::ADH1(t)*	This study
pGF-V1431	pRS316; *prCDC11::AcrIIA4(E66A Y67A)::ADH1(t)*	This study
pGF-V1432	pRS316; *prCDC11::AcrIIA4(N64A Q65A)::ADH1(t)*	This study

pGF-V1433	pRS316; *prCDC11::AcrIIA4(E49A V52A)::ADH1(t)*	This study
pGF-V1434	pRS316; *prCDC11::AcrIIA4(E45A E47A)::ADH1(t)*	This study
pGF-V1435	pRS316; *prCDC11::AcrIIA4(E40A Y41A)::ADH1(t)*	This study
pGF-V1436	pRS316; *prCDC11::AcrIIA4(D37A G38A)::ADH1(t)*	This study
pGF-V1437	pRS316; *prCDC11::AcrIIA4(T22A D23A)::ADH1(t)*	This study
pGF-V1438	pRS316; *prCDC11::AcrIIA4(D14A Y15A)::ADH1(t)*	This study
pGF-V1439	pRS316; *prCDC11::AcrIIA4(D5A E9A)::ADH1(t)*	This study
pGF-V1470	pRS316; *prCDC11::AcrIIA4(D14A)::ADH1(t)*	This study
pGF-V1471	pRS316; *prCDC11::AcrIIA4(N36A)::ADH1(t)*	This study
pGF-V1472	pRS316; *prCDC11::AcrIIA4(D37A)::ADH1(t)*	This study
pGF-V1535	pRS316; *prCDC11::AcrIIA4(G38A)::ADH1(t)*	This study
pGF-V1536	pRS316; *prCDC11::AcrIIA4(N39A)::ADH1(t)*	This study
pGF-V1473	pRS316; *prCDC11::AcrIIA4(E40A)::ADH1(t)*	This study
pGF-V1474	pRS316; *prCDC11::AcrIIA4(N48A)::ADH1(t)*	This study
pGF-V1475	pRS316; *prCDC11::AcrIIA4(D69A)::ADH1(t)*	This study
pGF-V1476	pRS316; *prCDC11::AcrIIA4(E70A)::ADH1(t)*	This study
pGF-V1477	pRS316; *prCDC11::AcrIIA4(E72A)::ADH1(t)*	This study
pGF-V1534	pRS316; *prCDC11::AcrIIA4(F73A)::ADH1(t)*	This study
pGF-V1478	pRS316; *prCDC11::AcrIIA4(D76A)::ADH1(t)*	This study
pGF-V1479	pRS316; *prCDC11::AcrIIA4(M77A)::ADH1(t)*	This study
pGF-V1480	pRS316; *prCDC11::AcrIIA4(D23R)::ADH1(t)*	This study
pGF-V1481	pRS316; *prCDC11::AcrIIA4(N39R)::ADH1(t)*	This study
pGF-V1482	pRS316; *prCDC11::AcrIIA4(D69R)::ADH1(t)*	This study

pGF-V1483	pRS316; *prCDC11::AcrIIA4(E70R)::ADH1(t)*	This study
pGF-V1484	pRS316; *prCDC11::AcrIIA4(Y67A D69A)::ADH1(t)*	This study
pGF-V1485	pRS316; *prCDC11::AcrIIA4(D69A E70A)::ADH1(t)*	This study
pGF-V1220[f]	pRS425; *prSNR52::Sp-sgRNA(u1-20WT)::SUP4(t)*	This study
pGF-425+IVL1277[g]	pRS425; *prSNR52::Sp-sgRNA(mCherry)::SUP4(t)*	[10]
pGF-IVL1431	pRS316; *prCDC11::GFP::AcrIIA4(E70A E71A)::ADH1(t)::Hyg*^{R}	This study
pGF-IVL1432	pRS316; *prCDC11::GFP::AcrIIA4(E40A Y41A)::ADH1(t)::Hyg*^{R}	This study
pGF-IVL1433	pRS316; *prCDC11::GFP::AcrIIA4(D14A Y15A)::ADH1(t)::Hyg*^{R}	This study
pGF-IVL1388[h]	pRS316; *prCDC11::AcrIIA2(2-11Δ)::ADH1(t)::Hyg*^{R}	This study
pGF-IVL1389	pRS316; *prCDC11::AcrIIA2(2-21Δ)::ADH1(t)::Hyg*^{R}	This study
pGF-IVL1392	pRS316; *prCDC11::AcrIIA2(114-123Δ)::ADH1(t)::Hyg*^{R}	This study
pGF-IVL1393	pRS316; *prCDC11::AcrIIA2(104-123Δ)::ADH1(t)::Hyg*^{R}	This study
pGF-IVL1390	pRS316; *prCDC11::AcrIIA4(2-11Δ)::ADH1(t)::Hyg*^{R}	This study
pGF-IVL1391	pRS316; *prCDC11::AcrIIA4::ADH1(t)::Hyg*^{R}	This study
pGF-IVL1394	pRS316; *prCDC11::AcrIIA4(78-87)::ADH1(t)::Hyg*^{R}	This study
pGF-IVL1395	pRS316; *prCDC11::AcrIIA4(68-87)::ADH1(t)::Hyg*^{R}	This study

[a]The sgRNA cassette was constructed as previous described [2, 11]. Briefly, 269 bp of the *SNR52* promoter sequence, 20 bp of the *SUP4* terminator sequence, and appropriate crRNA and tracrRNA (per orthologous species used) were included. A guide sequence of 20 bp was used.
[b]The anti-CRISPR AcrIIA2 and AcrIIA4 genes were synthesized *de novo* with a yeast codon bias and cloned into *CEN*-based yeast expression vectors under control of the *CDC11* promoter using *in vivo* plasmid assembly.
[c]GFP includes the substitutions F64L and S65T.
[d]eGFP includes the substitutions F64L, S65T, R88Q, and H239L.
[e]The general cloning scheme for AcrIIA2 and AcrIIA4 mutants included the following. First, the *prCDC11::AcrIIA2/A4::ADH1(t)* fragment was sub-cloned into a TOPO II vector (pCR-Blunt II-TOPO, Life Technologies, Inc.). Second, substitutions were introduced using PCR. Third, the entire construct was sub-cloned to the pRS316 vector using the flanking *NotI/SpeI* sites.

[f]The sgRNA(u1) includes the crRNA: 5’ CGGTGGACTTCGGCTACGTA 3’.
[g]The sgRNA(mCherry) includes the crRNA: 5’ CAAGGAGTTCATGCGCTTCA 3’.
[h]For constructs containing an N-terminal or C-terminal deletion, residues were removed during *in vivo* plasmid assembly.

Table S3: Comparison of AcrIIA4 substitution mutants—effects on Cas9 binding, *in vitro* inhibition of *Sp*Cas9, and *in vivo* inhibition of a Cas9-based gene drive.

**AcrIIA4 Residue(s)**[a]	**This study**	**Dong *et al.*, 2017 [12]**	**Yang and Patel, 2017 [13]**
D5, E9, N12	D5A/E9A, no effect on inhibition[b]	N12T, no effect on binding[c] or inhibition function[d]	ND[e]
D14, K18, E40	D14A, no effect on inhibition; D14A/Y15A[f], strong decrease in inhibition; E40A, no effect on inhibition; E40A/Y41A, very strong loss of all inhibition	D14R/E40R, slight decrease in binding, inhibition ND	D14A, minor/no loss of binding[g], partial loss of inhibition[h]; D14A/K18A, minor/no loss of binding, partial loss of inhibition; D14K, modest loss of binding, strong loss of inhibition; E40A, minor/no loss of binding, partial loss of inhibition; E40K, strong loss of binding, minor loss of inhibition
D23, N25, S26	D23R, very minor decrease in inhibition; T22A/D23A, no effect on inhibition	D23R, no effect on binding, decreased inhibition	D23A/S24A/N25A, minor/no loss of binding, partial loss of inhibition; D23K, minor/no loss of binding, minor loss of inhibition; N25K, minor/no loss of binding, modest loss of inhibition; S26K, minor/no loss of binding, strong loss of inhibition
N36	N36A, no effect on inhibition	N36Y, decreased binding, decreased inhibition;	ND

		N36R/D69R, decreased binding, inhibition ND	
D37, G38	D37A, G38A and D37A/G38A, all have no effect on inhibition	G38A, no effect on binding, decreased inhibition	ND
N39	N39A, minor decrease in inhibition; N39R, strong decrease in inhibition	N39R, decreased binding, decreased inhibition	N39A, minor/no loss of binding, moderate loss of inhibition; N39K, minor/no loss of binding, strong loss of inhibition
E40, Y41	E40A, no effect on inhibition; E40A/Y41A[f], very strong loss of all inhibition	D14R/E40R, slight decrease in binding, inhibition ND	E40A, minor/no loss of binding, partial loss of inhibition; E40K, strong loss of binding, minor loss of inhibition
E45, E47, N48, E49, V52, N64, Q65	E45A/E47A, N48A, E49A/V52A and N64A/Q65A, no effect on inhibition	ND	ND
E66, Y67, E68	E66A/Y67A, no effect on inhibition; Y67A/D69A, strong loss of inhibition; E68A/D69A, no effect on inhibition	ND	Y67A/D69A/E70A, strong loss of binding, strong decrease in inhibition; N39A/Y67A/D69A/E70A, near total loss of binding, strong decrease in inhibition; Y67K, moderate loss of binding, moderate loss of inhibition
D69	D69A, no effect on inhibition; D69R, minor decrease in inhibition; E68A/D69A, no	D69R, slight decrease in binding, decreased inhibition;	Y67A/D69A/E70A, strong loss of binding, strong decrease in inhibition;

	effect on inhibition; D69A/E70A, very strong loss of all inhibition	N36R/D69R, decreased binding, inhibition ND	N39A/Y67A/D69A/E70A, near total loss of binding, strong decrease in inhibition; D69K, minor/no loss of binding, strong decrease in inhibition
E70	E70A, strong loss of inhibition; E70R, very strong loss of inhibition; D69A/E70A, very strong loss of inhibition; E70A/E71A[f], strong loss of inhibition	E70R, decreased binding, decreased inhibition	Y67A/D69A/E70A, strong loss of binding, strong decrease in inhibition; N39A/Y67A/D69A/E70A, near total loss of binding, strong decrease in inhibition; E70K, moderate loss of binding, minor loss of inhibition
E71, E72, F73	E70A/E71A, strong loss of inhibition; E72A, no effect on inhibition; E72A/F73A and F73A, moderate loss of inhibition	ND	ND
Y74, N75, D76	Y74A/N75A and D76A, no effect on inhibition	ND	ND
M77	M77A and D76A/M77A, moderate loss of inhibition	ND	ND
Q78, T79, I80, T81, L82, K83, S84, E85, L86, N87	Q78A/T79A, I80A/T81A, L82A/K83A, S84A/E85A, and L86A/N87A, all show no effect on inhibition	ND	ND

[a]AcrIIA4 mutant substitutions are listed (in general) from residue 1 to 87. Groupings of more than one residue are for clarity: some combinations were only tested in combinations with other

residues. Several residues are found within multiple categories if they occur as part of double/triple/quadruple substitutions.

[b]The *in vivo* assay used in this study (Fig. 5) to provide a quantification of AcrIIA4 inhibitory function *S. pyogenes* Cas9 is by halting an active gene drive in diploid yeast after 5 hrs of nuclease induction.

[c]This study [12] used a GST pull-down assay to determine preloaded sgRNA-bound Cas9/AcrIIA4 binding (AcrIIA4 was fused to GST). Our interpretation of the amount of bound AcrIIA4 is from Extended Data Figure 3, part B, by comparing to control lanes.

[d]This study [12] utilized an *in vitro* DNA cleavage assay to illustrate the inhibitory effect of AcrIIA4 on Cas9. Our interpretation of the level of (remaining) inhibition of Cas9 nuclease activity is based Extended Data Figure 3, part C, by comparing to control lanes.

[e]ND, not determined.

[f]For several double mutant combinations, we tested the sgRNA loaded dCas9/AcrIIA4 association by an *in vivo* fluorescence localization assay (Fig. 6). All three mutants displayed a total loss of Cas9 association as determined by a lack of PM-localization.

[g]This study [13] performed an *in vitro* MBP-binding assay to Cas9 (AcrIIA4 fused to MBP). Our interpretation of the amount of bound AcrIIA4 is from Figures 3G and S3E by comparing to control lanes.

[h]This study [13] utilized an *in vitro* DNA cleavage assay using sgRNA-bound Cas9 and AcrIIA4. Our interpretation of the amount of AcrIIA4 inhibitory activity is from Figures 3H and S3F, by comparing to control lanes.

REFERENCES

1 Brachmann CB, Davies A, Cost GJ, Caputo E, Li J, Hieter P, *et al*. Designer deletion strains derived from *Saccharomyces cerevisiae* S288C: a useful set of strains and plasmids for PCR-mediated gene disruption and other applications. *Yeast* 1998;14(2):115-32.

2 Finnigan GC, Thorner J. mCAL: A new approach for versatile multiplex action of Cas9 using one sgRNA and loci flanked by a programmed target sequence. *G3* 2016;6(7):2147-56.

3 Kalderon D, Roberts BL, Richardson WD, Smith AE. A short amino acid sequence able to specify nuclear location. *Cell* 1984;39(3 Pt 2):499-509.

4 Finnigan GC, Thorner J. Complex *in vivo* ligation using homologous recombination and high-efficiency plasmid rescue from *Saccharomyces cerevisiae*. *Bio Protoc* 2015;5(13):e1521. http://www.bio-protocol.org/e.

5 Zheng L, Baumann U, Reymond JL. An efficient one-step site-directed and site-saturation mutagenesis protocol. *Nucleic Acids Res* 2004;32(14):e115.

6 Andersen MH, Graversen H, Fedosov SN, Petersen TE, Rasmussen JT. Functional analyses of two cellular binding domains of bovine lactadherin. *Biochemistry* 2000;39(20):6200-6.

7 Shao C, Novakovic VA, Head JF, Seaton BA, Gilbert GE. Crystal structure of lactadherin C2 domain at 1.7A resolution with mutational and computational analyses of its membrane-binding motif. *J Biol Chem* 2008;283(11):7230-41.

8 Christianson TW, Sikorski RS, Dante M, Shero JH, Hieter P. Multifunctional yeast high-copy-number shuttle vectors. *Gene* 1992;110(1):119-22.

9 Sikorski RS, Hieter P. A system of shuttle vectors and yeast host strains designed for efficient manipulation of DNA in *Saccharomyces cerevisiae*. *Genetics* 1989;122(1):19-27.

10 Roggenkamp E, Giersch RM, Wedeman E, Eaton M, Turnquist E, Schrock MN, *et al*. CRISPR-UnLOCK: multipurpose Cas9-Based strategies for conversion of yeast libraries and strains. *Front Microbiol* 2017;8:1773.

11 DiCarlo JE, Norville JE, Mali P, Rios X, Aach J, Church GM. Genome engineering in *Saccharomyces cerevisiae* using CRISPR-Cas systems. *Nucleic Acids Res* 2013;41(7):4336-43.

12 Dong, Guo M, Wang S, Zhu Y, Wang S, Xiong Z, *et al*. Structural basis of CRISPR-SpyCas9 inhibition by an anti-CRISPR protein. *Nature* 2017;546(7658):436-9.

13 Yang H, Patel DJ. Inhibition Mechanism of an Anti-CRISPR Suppressor AcrIIA4 Targeting SpyCas9. *Mol Cell* 2017;67(1):117-127.
